# An Unusual Case of an Acquired Aortopulmonary Fistula after Surgical Replacement of a Bicuspid Aortic Valve

**DOI:** 10.1155/2021/9088024

**Published:** 2021-09-23

**Authors:** Y. Khalid, N. Dasu, M. Daneshvar, P. Jang, A. Patel, K. Dasu, A. Shah

**Affiliations:** ^1^Department of Cardiology, The Wright Center for Graduate Medical Education, Scranton, PA, USA; ^2^Department of Gastroenterology, Jefferson Health New Jersey, Cherry Hill, NJ, USA; ^3^Department of Internal Medicine, Jefferson Heath New Jersey, Stratford, NJ, USA; ^4^Department of Internal Medicine, St. Mary's Medical Center, Langhorne, PA, USA; ^5^Department of Biomedical Sciences and Professional Studies, Drexel University, Philadelphia, PA, USA; ^6^Department of Cardiology, University of Pittsburgh Medical Center, Pittsburgh, PA, USA

## Abstract

Aortopulmonary fistulas are extremely rare and often occur as a result of long-standing aortic aneurysms. They are most frequently due to the erosion of a false aneurysm of the ascending or descending thoracic aorta into the pulmonary artery. Patients generally present with symptoms of acute decompensated heart failure due to a sudden formation of a left-to-right shunt. Here, we present the case of a 63-year-old male who acquired an aortopulmonary fistula four months after undergoing successful bioprosthetic aortic valve replacement.

## 1. Introduction

Infective endocarditis (IE) is a commonly seen diagnosis that is often managed with antimicrobial therapy. It can require surgical intervention in certain complicated cases. There are a number of indications for early surgical intervention including signs of heart failure, paravalvular extension of the infection with the development of an annular or aortic abscess, destructive penetrating lesions (i.e., fistula), persistent infection 5-7 days after starting antibiotic therapy, and difficult-to-treat infections [1]. Postoperative complications commonly include acute renal failure, prolonged mechanical ventilation, and tamponade. More rarely, there can be fistula formation not just between cardiac chambers, but also between the aorta and pulmonary artery leading to the formation of an aortopulmonary fistula.

Furthermore, an acquired aortopulmonary fistula is an extremely rare, but often fatal complication that requires prompt recognition and urgent surgical intervention. It can rapidly lead to cardiac tamponade or hemorrhagic shock. The majority of aortopulmonary fistula cases have been diagnosed post mortem and extremely rarely in patients who are alive [2]. Case studies have shown its associations with infective endocarditis (IE) and valvular surgery as a result of thoracic aortic aneurysm formation [3–5]. In the following case report, we discuss the rare, but important, complication of an aortopulmonary fistula formation following a bioprosthetic surgical aortic valve replacement (SAVR) for treatment of an aortic valve endocarditis with aortic root abscess.

## 2. Case Presentation

**Figure 1:**
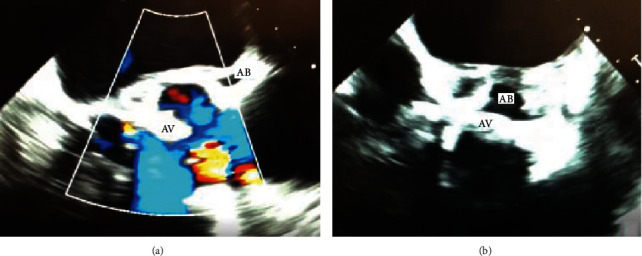
(a, b) Transesophageal echocardiogram showing aortic valve endocarditis (AV) and associated aortic root thickness and abscess. View in (a): color Doppler, apical long axis. View in (b): short-axis.

**Figure 2:**
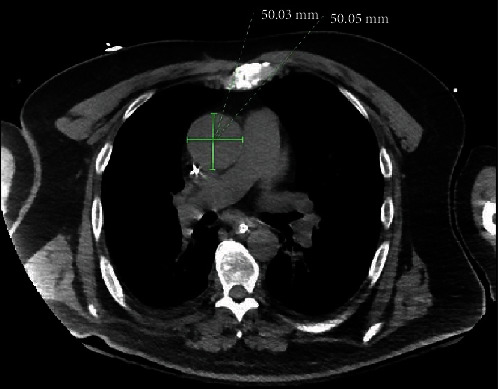
Soft-tissue axial view of CT chest demonstrating dilated aortic root.

A 63-year-old male with a past medical history of hypertension, hyperlipidemia, and obstructive sleep apnea initially presented with altered mental status and was admitted for septic shock secondary to methicillin-sensitive *Staphylococcus aureus* bacteremia and NSTEMI with troponin level up to 40 ng/mL. His cardiopulmonary exam was unremarkable on the initial exam. Due to poor images on a transthoracic echocardiography (TTE), a transesophageal echocardiography (TEE) and soft tissue chest CT scan was also performed which revealed a severely stenotic bicuspid aortic valve with fusion of the left and noncoronary cusps as well as an ascending aorta dilatation of 4.8 cm and aortic root dilatation of 4.5 cm with concern for an aortic root abscess (Figures 1(a), 1(b), and 2). The patient's hospital course was complicated by an embolic stroke and embolic phenomenon of his right thumb, requiring stabilization prior to surgical intervention. The patient underwent an uncomplicated bioprosthetic aortic valve replacement with a 23 mm Magna Ease Tissue valve, a patch exclusion of the left coronary sinus abscess, and aortoplasty. A coronary artery bypass grafting with a saphenous vein graft to the left anterior descending (LAD) artery was also performed, as the abscess had involved the LAD artery as well. The final pathological report demonstrated thrombotic vegetations on the valve leaflets consistent with acute endocarditis. Once stabilized, the patient was discharged to acute rehabilitation on a 6-week course of IV nafcillin based on culture sensitivities.

**Figure 3:**
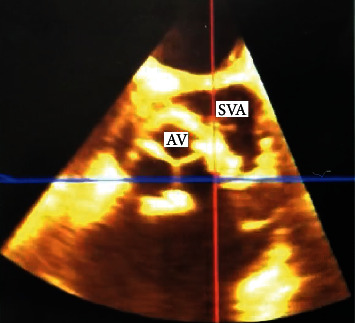
Transesophageal echocardiogram showing aortic sinus of Valsalva aneurysm extending posteriorly to interatrial septum (white arrow) and ventricular septal defect (yellow arrow). View: color Doppler, apical four chamber.

**Figure 4:**
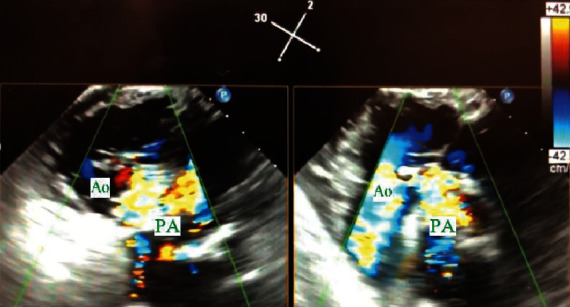
Transesophageal echocardiogram showing aortopulmonary fistula. View: color Doppler, long axis view—upper esophageal. AO: ascending aorta; PA: pulmonary artery.

About four months after discharge, the patient presented to an outpatient cardiology clinic for a routine postoperative follow-up. At that time, he was asymptomatic and reported having completed the antibiotic course. Physical exam, however, was notable for a left lower sternal harsh holosystolic murmur. He underwent stat TTE, which revealed an abnormal aortic valve with a possible leak near the patch. Upon arrival back to the hospital, TEE revealed a normally seated bioprosthetic aortic valve with no abnormal valvular motion or apparent dysfunction but had a large ventral septal defect (VSD) involving the lower border of the valve which entered the right ventricle adjacent to the septal leaflet of the tricuspid valve (Figure 3). It also showed an aortopulmonary artery fistula connecting the sinus of Valsalva to the pulmonary artery and moderate dilatation of the aortic root with an aneurysmal appearance (Figure 4). Given the level of complexity of the case, he was transferred to a tertiary care facility, at which he successfully underwent freestyle aortic root (23 mm) and ascending aortic replacement (24 mm), reimplantation of SVG to LAD bypass graft, and VSD closure.

## 3. Discussion

For surgical management of infective endocarditis, bioprosthetic SAVR is a successful technique with normally minimal complications as compared to other modalities [6, 7]. Aortopulmonary artery fistula formation following bioprosthetic SAVR is an extremely rare but potentially lethal complication of the procedure. Aortopulmonary fistulas can form secondary to infective endocarditis fistulation, thoracic aortic aneurysm rupture, a ruptured sinus of Valsalva aneurysm, or as a complication of procedures performed on the aorta or pulmonic valve. In the present case, the patient had an extensive paravalvular infective endocarditis that formed from an aortic root aneurysm which required aortic valve replacement surgery as a result. Additionally, the patient's blood cultures identified *Staphylococcus* as the causative organism of the infective endocarditis, which has been shown to be the most common organism associated with any type of fistula formation [5]. Though it is hard to differentiate the main cause that led to the development of the fistula, the negative blood cultures and absence of systemic symptoms helped us rule out a recurrent infection. Therefore, the fistula formation was determined to likely be a complication of the valve replacement surgery.

Additionally, a ruptured sinus of Valsalva aneurysm can result in an aortopulmonary fistula formation [7]. A sinus of Valsalva aneurysm is an enlargement of the aortic root area between the aortic valve annulus and the sinotubular ridge. It is found in roughly 0.09% of the general population, and it has been associated with endocarditis [8]. An aortic root aneurysm was identified in the present case, and we believe that the surgical intervention on the right sinus of Valsalva aneurysm led to the development of the aortopulmonary fistula and possibly also to the ventricular septal defect. However, of note, postoperative echocardiography was not completed. Thus, the VSD could have been iatrogenic, as it is a rarely known complication following surgical repair of cardiac valves. A postoperative echocardiography is of utmost importance as it would have elucidated the VSD.

Furthermore, although there were no clinical signs or symptoms of right heart failure, right ventricular overload was evident on echocardiogram. Given the large size of the ventricular septal defect and the aortopulmonary fistula, the patient would be expected to show symptoms of right heart failure, such as dyspnea, hemoptysis, chest pain, orthopnea, or syncope. Interestingly, the patient had no symptoms when the structural defects were identified at routine follow-up. This demonstrates how imperative follow-up visits are for this patient population given that an aortopulmonary fistula can present without symptoms and an early and accurate diagnosis can be life-saving. It also highlights the importance of obtaining postoperative echocardiography. [2].

## 4. Conclusions

Iatrogenic ventricular septal defect and aortopulmonary artery fistula are extremely rare and lethal complications of bioprosthetic SAVR and reconstruction in the setting of acute infective endocarditis. These fistulas can lead to rapid deterioration of the patient and so early and accurate identification is crucial to the survival of the patient. However, these fistulas can initially present asymptomatically, and so, close follow-up after discharge must be emphasized and encouraged for all patients. Future studies could reevaluate follow-up guidelines in this patient population to better prevent such devastating results.
